# Draft Genome Sequence of *Streptococcus orisasini* SH06, Isolated from a Healthy Thoroughbred Gastrointestinal Tract

**DOI:** 10.1128/genomeA.01566-15

**Published:** 2016-01-14

**Authors:** Misako Takagi, Akiyo Nakano, Hidehiro Toh, Kenshiro Oshima, Kensuke Arakawa, Fumihiko Nakajima, Kosuke Tashiro, Tekefumi Kikusui, Fujitoshi Yanagida, Hidetoshi Morita

**Affiliations:** a The Institute of Enology and Viticulture, University of Yamanashi, Kofu, Yamanashi, Japan; b Crossfield-Bio Inc., Tama, Tokyo, Japan; c Department of Microbiology and Infectious Diseases, Nara Medical University, Kashihara, Nara, Japan; d Medical Institute of Bioregulation, Kyushu University, Fukuoka, Japan; e Graduate School of Frontier Sciences, The University of Tokyo, Kashiwa, Chiba, Japan; f Graduate School of Environmental and Life Science, Okayama University, Okayama, Japan; g Northern Farm, Yufutsu-gun, Hokkaido, Japan; h Graduate School of Systems Life Sciences, Kyushu University, Fukuoka, Japan; i School of Veterinary Medicine, Azabu University, Sagamihara, Kanagawa, Japan

## Abstract

*Streptococcus orisasini* SH06 was isolated from a healthy thoroughbred gastrointestinal tract. Here, we report the draft genome sequence of this organism. This paper is the first published report of the genomic sequence of *S. orisasini*.

## GENOME ANNOUNCEMENT

Horses have a large and complex gastrointestinal tract. We have previously isolated lactobacilli from feces of nine thoroughbreds (1). During this work, we have also isolated coccoid forms of bacterium (strain SH06) from a healthy thoroughbred (male, 4-year-old). We performed 16S rRNA gene sequencing and thus confirmed that strain SH06 belongs to *Streptococcus orisasini*, which was reported as a novel species in 2013 (2). We deposited *S. orisasini* SH06 in the Japan Collection of Microorganisms (JCM) as strain JCM 17305.

The *S. orisasini* SH06 genome was paired-end sequenced using Illumina’s MiSeq platform. Genomic libraries containing 600 to 1,000 bp inserts were constructed and sequenced, yielding 3,509,312 sequences that provided 346-fold coverage from both ends of the genomic clones. The sequence reads were assembled using Newbler version 2.8 (Roche), and the assembled genome consists of 67 contigs with a total length of 2,299,160 bp. The draft genome of *S. orisasini* SH06 contained 2,099 predicted protein-coding genes, most of which shared high sequence homology to those of *Streptococcus mutans*, which is commonly found in the human oral cavity. The type strain of *S. orisasini* is isolated from the oral cavity of donkeys (2). Thus, *S. orisasini* might be a member of horse oral microbiota.

Several *Streptococcus* species, such as *S. equi*, *S. bovis*, *S. zooepidemicus*, *S. dysgalactiae* subsp. *equisimilis*, *S. pneumoniae* (capsule type III), *S. henryi*, and *S. caballi*, have been isolated from the horse gastrointestinal tract as pathogens (3–7). On the other hand, *S. orisasini* SH06 is likely to be a nonpathogenic strain, because this strain was isolated from a healthy thoroughbred gastrointestinal tract. Thus, the genome information of this species will be useful for further studies of the pathogenicity of *Streptococcus* for equine species.

### Nucleotide sequence accession numbers.

The draft genome sequence for *S. orisasini* SH06 (= JCM 17305) has been deposited in the DDBJ/GenBank/EMBL database under the accession numbers BCLE01000001 to BCLE01000067.
